# Amoxicillin separation from pharmaceutical wastewater by high permeability polysulfone nanofiltration membrane

**DOI:** 10.1186/2052-336X-11-9

**Published:** 2013-06-13

**Authors:** Reza Derakhsheshpoor, Maryam Homayoonfal, Ahmad Akbari, Mohammad Reza Mehrnia

**Affiliations:** 1 School of Chemical Engineering, College of Engineering, University of Tehran, Tehran, Iran; 2 Institute of Nanoscience and Nanotechnology, University of Kashan, Kashan, Iran

**Keywords:** Amoxicilin separation, Polysulfone membrane, High permeability nanofiltration, UV-grafting technique, Polyethylene glycol

## Abstract

In this study, high permeability flat sheet polysulfone nanofiltration membranes were prepared for amoxicillin (AMX) recovery from pharmaceutical wastewater. Membrane fabrication includes two steps: raw ultrafiltration membrane synthesis by phase inversion method and nanaofiltration membrane synthesis by surface photopolymerization. Raw ultrafiltration membranes were synthesized using different molecular weights of polyethylene glycol (PEG) as pore former and different coagulation bath temperatures (CBTs). The synthesized ultrafiltration membranes were modified using UV-assisted polymerization technique and their performance in the separation of AMX at different pHs, were studied. The results showed that the more irradiation time, the smaller surface pore size. Moreover, the membranes made with higher molecular weight of PEG and coagulation bath temperatures were more susceptible for UV-modification at these conditions; fabricated membranes had higher flux as well as relatively high AMX separation. Moreover, pH enhancement increased AMX rejection by 85%. The effect of irradiation on membrane surface morphology was studied by SEM surface images and the morphological effects of pore former and coagulation bath temperatures on membrane structure were confirmed by SEM cross section images. A fairly comprehensive discussion about the effects of PEG, coagulation bath temperature and irradiation time on membrane structure and AMX recovery performance was represented in this study.

## Background

Antibiotics have transformed infectious diseases treatment to play an important role in improving health, reducing disease and mortality. Based on data given by Moreno-Bonbi et al. [1], total consumption of antibiotics in the world is estimated over than 100 to 200 ktons per year. According to Zucker and Levy report (2009), Iran is on the highest rank in antibiotic consumption and this matter has increased antibiotic resistance in Iran [2]. Because of the widespread use of antibiotics, these substances were found in various environmental samples such as wastewater, groundwater and rivers, hospital liquid waste, soil and manure [3]. The important issue about the effects of antibiotics on the environment, is the development of antibiotic resistance which is considered a threat to the usefulness of antibacterial substances.

Persistence of pharmaceutical residues was demonstrated for common treatments applied in drinking water treatment plants (DWTPs), such as sand filtration [4], chemical coagulation/flocculation [5,6], chlorination [7], ultraviolet (UV) radiation [8], ozonation, advanced oxidation processes (AOP) and activated carbon [4]. Also, techniques that have been gaining attention in the past few years are pressure-driven membrane processes nanofiltration (NF) and reverse osmosis (RO). These two treatments seem to be able to effectively remove most organic and inorganic compounds and microorganisms from raw water [9,10] and their application in drinking water treatment has been the focus of attention of many researchers [11].

Membrane separation mechanisms of organic compounds, has been investigated usually by removing pesticides from water and wastewater. A qualitative rejection diagram for organic micropollutants during membrane treatment has been presented to predict membrane separation efficiency based on solute and membrane properties [12]. Based on these findings, the major factor in the separation of organic compounds is relative size of solute molecule to membrane pores. On the other side if the organics in solution appeared to be charged, Donan exclusion mechanism could also play a role in the separation [12-14]. Most researchers who accomplished membrane for pharmaceuticals component separation, especially antibiotics, used commercial membranes with polyamide active layer [11,15-19]. There is a general notion that nonionazable organic solutes of molecular mass ranged between 200 and 300 g/mol are efficiently rejected by the NF/RO membranes and a minimum retention of 85% was obtained [11,16]. Verliefde et al. probed the effective parameters in separation of pharmaceutical with different molecular size, charge, and hydrophobic properties [15]. Nghiem and Hawkes inspected the effect of fouling on three nanofiltration membrane performances and found that depending on the size of membrane pores and the pH, fouling can have different effects on retention [19]. Kosutic et al. (2007) stated steric hindrance is the main mechanism in separation of uncharged solutes by studying separation of several antibiotics and recommended use of a tight NF membrane, due higher flux and comparable retention with respect to RO [16]. Zazouli et al. (2009) studied the effect of solution chemistry on the separation of some pharmaceuticals by two NF membranes [17]. They changed pH, ionic strength and added natural organic matter, as a strong foulant. The permeate flux would decrease with decreasing the pH solution and increasing ionic strength [17], therefore for charged surface membrane, surface properties and solution chemistry can affect on membrane efficiency.

Whereas the energy consumption per volume of produced permeate may be a significant portion of the cost of filtration, fabrication of membrane for a specific separation with high permeability has great economical significance. Therefore the main aim of this research is fabrication of a high permeability negative charged NF membrane for amoxicillin separation from water. Since one of the important advantages of nanofiltration is exploitation of electrostatic repulsion mechanism in separation, in this study, acrylic acid bounded to membrane and membranes with charged surfaces were developed. Presence of this material not only increases surface hydrophilicity which increases membrane permeability, but also heightens membrane surface charge and so increases AMX recovery. Our previous work [13] showed that these membranes have high efficiency in the separation of metallic ions from water so we expect the synthesized membrane would be able to separate amoxicillin -with higher MW and surface charge than metallic ions- from water.

## Materials and methods

### Materials

Polysulfone (PSf, MW75000 Da) as main polymer matrix, was supplied by Acros Organics. N-methylene-2-pyrrolidone (NMP) supplied by Merck and used as a solvent for PSf polymer solution preparation without further purification. Poly(ethylene glycol) with average molecular weight from 400 to 10000 Da, acrylic acid and other chemicals were purchased from Merck company. Pure amoxicillin powder, were supplied by Dana Pharmaceutical Company (Iran). The main characteristics of amoxicillin are listed in Table 1.

**Table 1:** Characteristics of amoxicillin molecule

**Characteristic**	**Amount**
Trade names	Actimoxi, Alphamox, Amocla,Tycil, Amoxil, Trimox
Systematic (IUPAC) name	2*S*,5*R*,6*R*)-6-{[(2*R*)-2-amino-2-(4-hydroxyphenyl)-acetyl]amino}-3,3-dimethyl-7-oxo-4-thia-1-azabicyclo[3.2.0]heptane-2-carboxylic acid
Chemical Structure	
Formula	C_16_H_19_N_3_O_5_S
Mol. mass	365.4 g/mol
State	Solid
Melting Point	194°C
Solubility in water	mg/L 3430
pKa	9.53,7.31,2.4

### Membrane preparation and modification

Due to the fact that fabrication of nanofiltration membrane usually is a two stages process, here, we prepared an UF membrane via phase inversion method then aided UV- assisted polymerization method to deposit a thin layer on the UF sub layer and produce NF membrane. After membrane synthesis, sub layer membrane construction parameters such as pore forming agent molecular weight and coagulation bath temperature and top layer membrane construction such as time of photopolymerization investigated.

Casting solution was prepared by blending of PSf/PEG/NMP as 17/8/75 in wt%. After blending the mixture was stirred (magnetic stirrer, Heidolph MR Hei- Standard, Germany) for 24 h at 75°C. Then ultrasonic stirrer was used for 40 min. Then it was cooled to room temperature. The solution was cast uniformly on clean glass plate using a hand-casting knife with a gap set at 300 μm and then immersed in reverse osmosis water coagulation bath at certain temperature for 24 h [20-25].

For preparation of nanofiltration membranes from the ultrafiltratiom membranes obtained during phase inversion step, UV-assisted polymerization of acrylic acid was accomplished on raw membrane. This technique increases membrane wettability and decreases pore size [13,14]. Irradiation equipment contains a rotating cylinder, which membrane is placed on its peripheral surface to immerse periodically in 6% wt. acrylic acid monomer solution and to be irradiated by a 125W UV lamp. When the cylinder continues rotating, wetted membrane is exposed UV irradiation and polymerization reaction implement on membrane surface. This process continues for a certain time.

Different membranes were made by use of different molecular weight of PEG in dope solution, coagulation bath temperatures and irradiation times during modification (see Table 2). In this research, by inspection of coagulation bath temperature on performance of PEG-400, PEG-1500, PEG-3000, PEG-6000 membranes, high temperature coagulation bath and high molecular weight of PEG diagnosed favorable. In next stage, we used longer irradiation times in modification step to compensate rejection decline due to high molecular weight PEG utilization.

**Table 2:** Primary experiments plan

**Membrane name**	**PSf wt%**	**NMP wt%**	**PEG**		**CBT (°C)**	**Irradiation time (Min)**
			**Type**	**Wt%**		
PEG-400	17	75	400	8	22,40,60	0,10,20,30
PEG-1500	17	75	1500	8	22,40,60	0,10,20,30
PEG-3000	17	75	3000	8	22,40,60	0,10,20,30
PEG-6000	17	75	6000	8	22,40,60	0,10,20,30

### Membrane filtration performance

**Figure 1:**
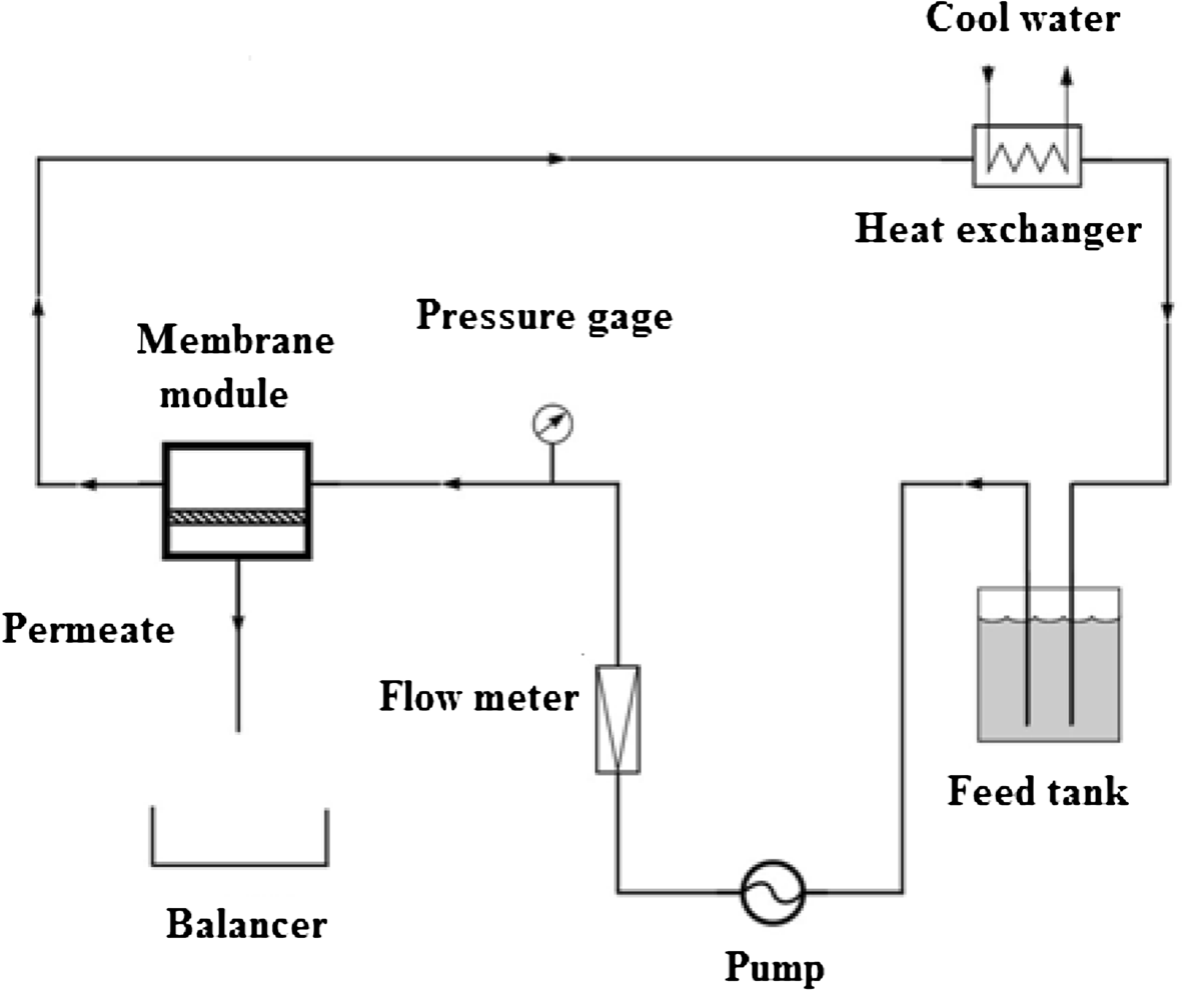
The schematic illustration of crossflow filtration system.

All experiment were done at room temperature (25±2°C) and a pressure of 3 bar using a crossflow filtration system [20-25] with 32 cm^2^ effective area cell. Membranes were pressurized at 4.6 bar before application in experiments. The schematic illustration of cross flow filtration system is shown in Figure 1.

### Membrane characterization

Since the main portion of amoxicillin loss during production process occurs in crystallization step, in which amoxicillin concentration is approximately 100 mg/l, so feed concentration were adjusted at 100 mg/L. considering that feed solution density is about 1, by measuring permeate weight at certain times membrane permeability, were calculated by equation 1:

(1) $$L_{p}=\frac{l}{A\Delta t\Delta p}$$

In which L is permeate volume (liter), A is the membrane surface area (m^2^), Δt is the permeation time (h) and ΔP is transparent pressure on membrane sides (bar). In order to describe the behavior of membrane separation, retention of solute were determined by measuring the concentration of amoxicillin in the feed and permeate streams as below:

(2) $$R\%=\left(1-\frac{c_{p}}{c_{f}}\right)\times 100$$

In which Cp and Cf are amoxicillin concentration in permeate and feed solution. Amoxicillin concentration measured by spectrophotometer (GBC, model Cintra 101, Australia ) at 229 nm wavelength [18].

Molecular weight cut off of a membrane refers to the MW of a neutral solute having a rejection beyond 90%. Solutes generally are neutral and have MWs spanning the range of proteins, dextran and PEG. Here MWCO is based on the rejection of PEG. The analytical method for determining PEG concentration is given by Sabde et al. [26]. PEG solutions were analyzed using a UV–Visible spectrophotometer (GBC, model Cintra 101, Australia) at a wavelength of 535 nm. The membrane mean pore size was determined according to the equation reported by Causserand et al. [27].

SEM image was taken by SEM microscope (Philips XL30, Germany) after complete sample preparation i.e. sample freezing by liquid nitrogen, breaking sample and gold scattering.

The chemical structure of a membrane changes after photo polymerization. Therefore the efficiency of UV-grafting reactions were checked by FTIR in transmission mode (ATR) using the Nicolet Magna IR 550. The spectra of unmodified and modified membranes were compared.

## Results

The point should be mentioned here is that all experiments were done base on Table 2. Because of same trends best results (highest membrane performance) were represented here to describe effect of one parameter i.e. high CBT were selected to show effect of irradiation time.

### Effect of PEG molecular weight and coagulation bath temperature

**Figure 2:**
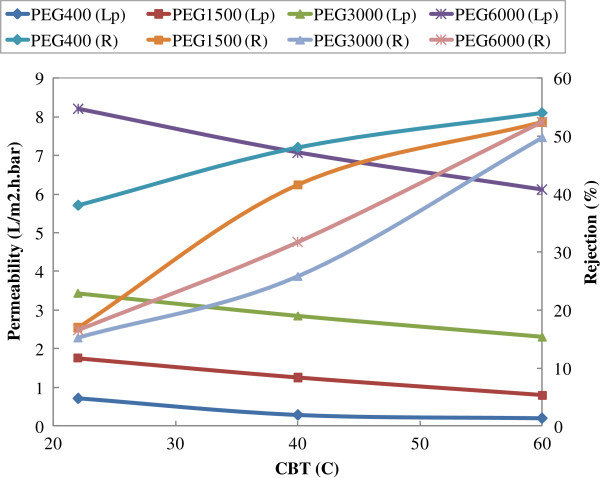
Effect of CBT and PEG molecular weight on permeability and AMX recovery (irradiation time=30 min; pH=8.3).

The effects of CBT and PEG molecular weights on permeability and AMX recovery are shown in Figure 2.

**Figure 3:**
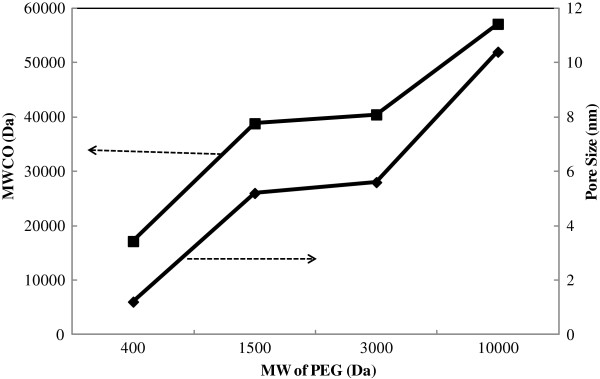
Effect of PEG MW on pore size and MWCO of UF membrane.

As seen from the figure, the increase in CBT deduces to increment in AMX recovery and decrement in AMX flux. On the other side, decrease in PEG MW has the similar effect to CBT on membrane performance. As the overall statement increase in CBT and decrease in pore former MW result in a membrane with the lesser porosity. This behavior has been confirmed by measuring MWCO of membrane. Effect of MW of PEG on MWCO of synthesized ultrafiltration membrane is shown in the Figure 3.

As seen from the figure increase in MW of PEG from 400 to 1000 Da, increases MWCO of UF membrane from 6 to 52 kDa.

### Effect of pH on membrane performance

**Figure 4:**
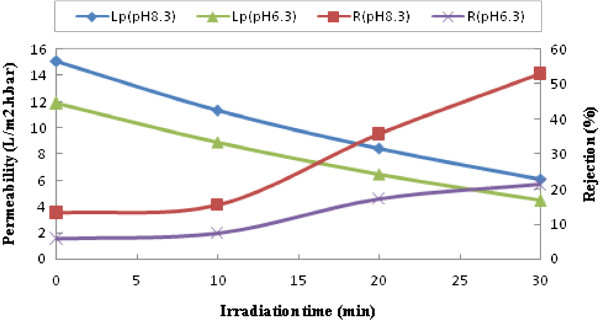
Effect of pH on PEG6000 membrane permeability and rejection in different irradiation times (CBT=60).

In this study, acrylic acid molecules placed on membrane surface. Since dissociation constant of this acid is about 4.3, membrane surface will be negative at neutral and basic pH. In case of charged membranes, pH plays an important role on membrane performance. Separation difference of various modified membranes at these two pH levels is shown in Figure 4.

As seen from the figure, with increasing modification time rejection and also, rejection differences of two distinct pH increase. This behavior shows that with increasing time of modification and binding more acrylic acid, membrane performance become more sensitive to pH. Furthermore, it is simply obvious from the figure that increase in feed pH from 6.3 to 8.3 improves 30% AMX recovery.

### Fabrication of high flux nanofiltration membrane

**Figure 5:**
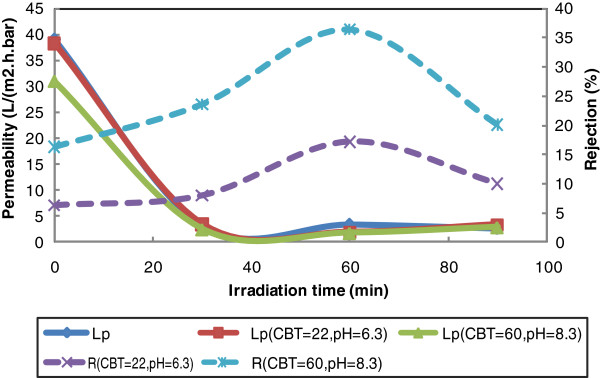
Effect of irradiation time on performance of membranes containing high-molecular-weight PEG, pH=8.3.

As the overall result higher surface charge and smaller pores at longer irradiation times increased AMX retention to an appropriate level. Figure 5 shows the effect of modification time on membrane permeability and AMX rejection.

**Figure 6:**
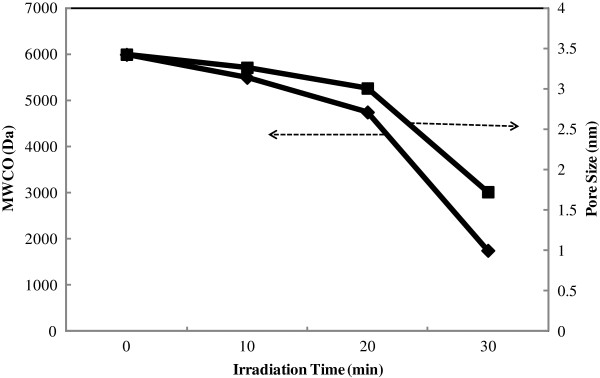
Effect of irradiation time on MWCO and pore size of modified membrane.

By increase in irradiation time pores shrunk and AMX recovery increased. Experiments showed increase in irradiation time from 60 to 90 minutes for PEG-10000 membrane leads to 43% rejection decrement at CBT=60°C. This performance is an evidence of decreasing in membrane pore size. Figure 6 shows the effect of increasing in irradiation time on membrane pore size.

Result of MWCO analysis showed that increase in irradiation time deduces to increase in deposition of a poly acrylic layer on membrane surface and so decrease in membrane pore size and MWCO.

### FTIR spectrum

**Figure 7:**
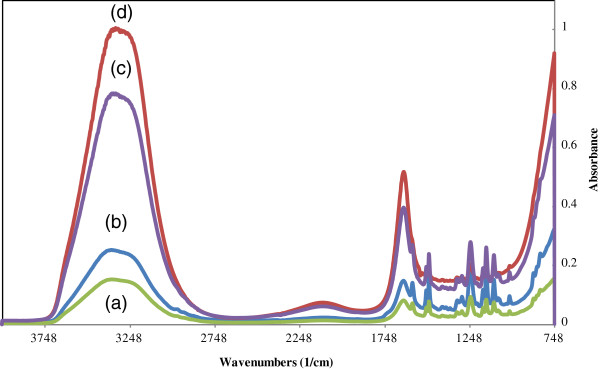
FTIR spectroscopy of nanofiltration membrane grafted at different irradiation time (a: 0 min; b: 10 min; c: 20 min; d: 30 min).

The effect of photopolymerization on the membrane surface was checked by FTIR spectroscopy. The results are shown in Figure 7.

It is clear from the figure that with increase in irradiation time, absorbance peak at 3396/cm and another at 1732/cm is intensify.

### Membrane morphology

#### SEM cross section images

**Figure 8:**
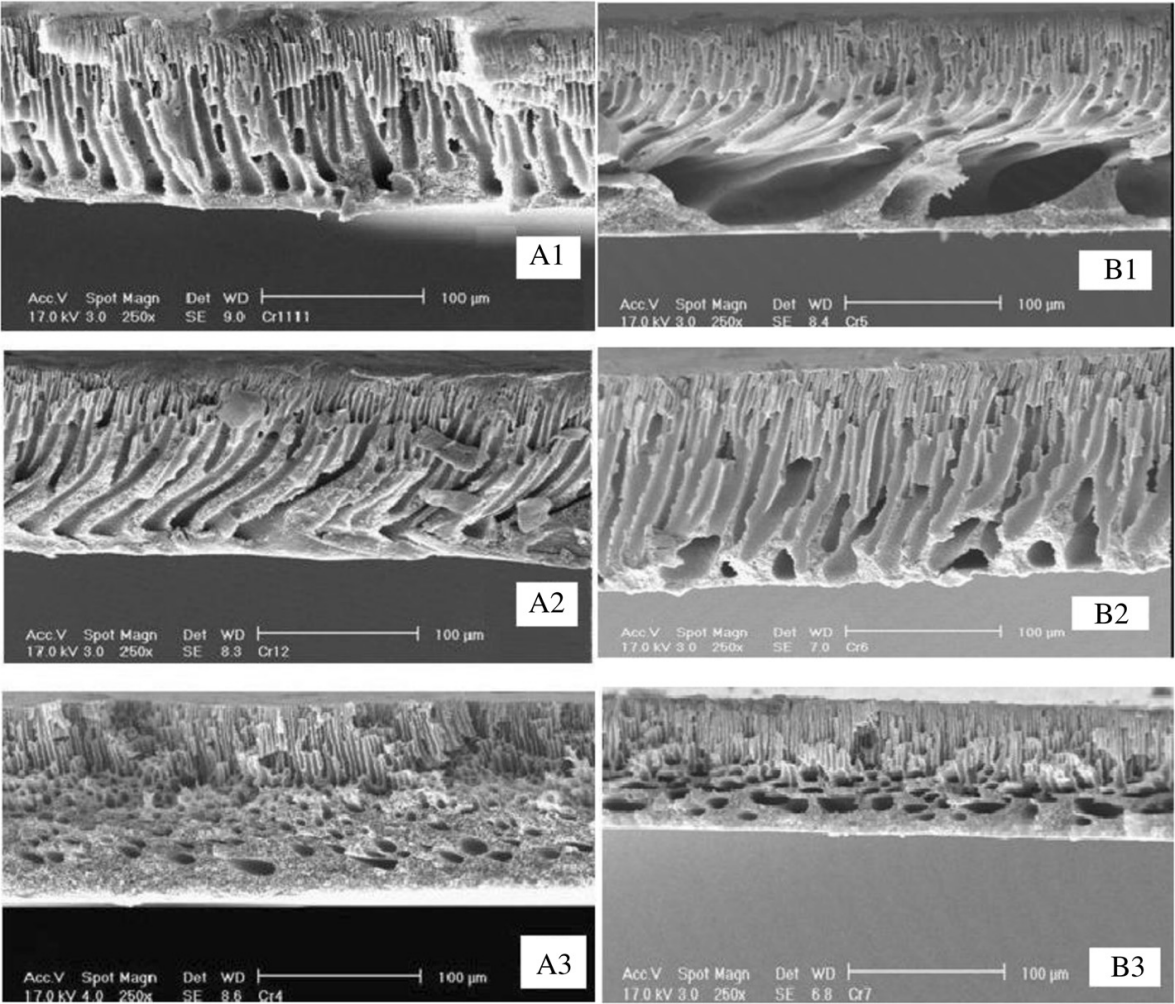
Effect of coagulation bath temperature (1:22; 2:40; 3:60°C) on A:PEG-400 and B: PEG-6000 membranes.

Variation in membrane cross-section by change in coagulation bath temperature for two types of membranes with different PEG molecular PEG is shown in Figure 8. As shown in SEM cross sectional images, coagulation bath temperature not only lead to smaller pores but also changes support layer structure from finger like (A1, B1) to sponge structure (A3, B3).

#### SEM surface images

**Figure 9:**
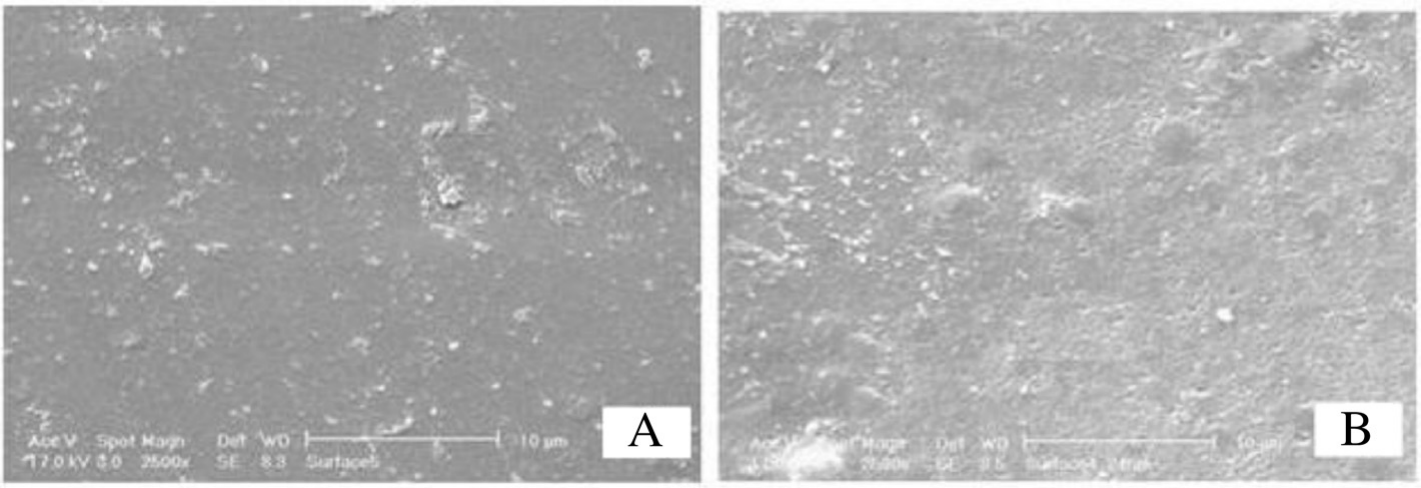
Effect of excessive radiation on PEG-10000 membrane: A: 60, B: 90 min irradiation.

Figure 9 shows membrane surface changes under extra radiation. As seen from figure, surface roughness decreases by increasing in irradiation time, therefore is an evidence of decreasing in pore size.

## Discussion

The effect of PEG molecular weight on permeability and AMX recovery is shown in Figure 2. PEG is known as pore forming agent in phase inversion method which increases pore size [22-25]. But some others expressed PEG can acts as pore reducing agent [20,21]. This behavior was mostly seen by low molecular weight PEG, i.e. 200 and 400 Da. It seems that prediction whether PEG acts as pore forming or reducing agent depends on the mechanism that PEG forms pores. If large amount of PEG void to coagulant i.e. water during immersion precipitation step, it will act as pore reducing agent in which PEG acts as nonsolvent because presence of this compound in dope solution leads to decrease in coagulation value [22].

In other words, PEG outgoes decrease net nonsolvent inflow and diffusion rate that causes denser structure and smaller pores. This behavior is imaginable by low molecular weight of polyethylene glycol, or a diluted polymer solution. But if the most of PEGs remain in polymeric film while coagulation, due to PEG hydrophilicity, the inlet rate of coagulant will heighten leading to increase the size of the pores. This manner is expected for higher molecular weight PEGs and more concentrated polymer solution. Because of this two different mechanism Chakrabarty et al. (2008) in contrary to other researchers observed decrease in pore size by increasing PEG molecular weight [23,24]. Here at a given coagulation bath temperature (as seen from Figure 2), by increasing PEG molecular weight permeability increased and rejection decreased as expected [13,22,25]. This behavior confirms by measuring MWCO of membrane. As is illustrated in Figure 3, the MWCO of membranes increases with increasing in MW of pore former (PEG). In fact increase in MW of PEG deduced to instantaneous demixing and fabrication of a membrane with larger pore size having high molecular weight cut off.

As can be seen from Figure 2, membrane permeability decreases and AMX recovery increases with increasing coagulation bath temperature. This is contrary to the trend reported by other researchers [28,29]. Of course, the polymer type and composition of polymeric solution at those studies are different from the membrane composition in this research. Kim and Lee (2003) stated decrease in diffusion rate lead to formation a membrane with a smaller pore size and a denser skin layer [30].

Reduction of membrane flux (permeability), due to temperature rise, could be also described based on thermodynamic laws governing the manufacture of the membrane. For multicomponent mixtures, demixing region becomes smaller at higher temperatures and as a result polymeric solution should take longer path to reach dimixing boundaray which cause delayed demixing and lead to smaller pores [31]. It seems that in lowest temperature PEG-400 membrane rejection is significantly higher than others. Since pore size decreases while using lower PEG molecular weight so higher retention was obtained. Furthermore membranes pores decreased by increase in bath temperature and solute retention improved. The noticeable point is that when using higher molecular additive, the pore size increased. Subsequently monomers potency increase to enter the pores and make pores inside charged [13]. So by increase in PEG molecular weight membrane permeability increase but rejection does not drop significantly [32].

Change in pH can increase or decrease membrane surface charge (see Figure 4). Even at a certain point membrane surface can be neutral which is nominated isoelectric point. If solute charge change in different pHs as seen for AMX [32], the effect of pH on solute separation becomes more important [33]. Based on acrylic acid dissociation constant (pKa=4.3), the membrane surface has negative charge, therefore, if in this pH range solute appeared negative, high rejection would expect due electrostatic repulsion between solute and membrane. As stated Elmolla and Chaudhuri (2010), amoxicillin is an amphoteric substance with pK_a1_=2.4, pK_a2_=7.4 and pk_a3_=9.6 [32]. Amoxicillin at medium pHs is zwitterion, at pH=2 mainly is cation and pH above 7.4 is anion. AMX has a great dependence of pH and is very high degradable at very low and very high pHs [34]. Considering above points two pH points were selected (6.3 and 8.3). The results showed that the increasing in irradiation time improves AMX recovery of nanofiltration membranes. This behavior is because of increasing in acrylic acid deposition (confirmed by FTIR spectrum Figure 7) which increases surface negative charge density and decreasing in membrane pore size (confirmed by MWCO analysis Figure 6) that accordingly improvement charge repulsion and AMX recovery. Separation of amoxicillin by commercial nanofiltration membrane has been reported by other researchers [35,36]. Photographted membranes considering MWCO data, have smaller pore size and considering FTIR analysis have more charge density than unmodified membrane.

The other effect that repulsion between solute and membrane causes, is lower tendency to fouling. Because repulsion existence between solute and membrane, solute molecules ability to get closer the membrane and deposit on membrane surface decreases which result in increased membrane permeability and reduced costs. As indicated, at higher pH, because amoxicillin converts anionic form, electrostatic repulsion between solute and membrane occurs and membrane permeability decreases and rejection increases. In fact in the lower pH (6.3), molecular sieve mechanism is dominate and results in medium rejection while Donnan repulsion mechanism is dominant for our photografted membranes at higher pH (8.3), an increase in pH have a great effect on amoxicillin recovery improvement. Moreover the membrane performance sensitivity to pH increases in longer irradiation time (more than 20 min) due to more density of acrylic acid (negative charge) on membrane surface.

According to above mentioned results, higher molecular weight PEG and lower bath temperature produce a membrane with larger pore size and is favorable for UV irradiation [13]. Therefore a membrane containing higher MW of PEG was chosen, modified during different irradiation times by UV assisted photopolymerization method [13,14,33,37], analyzed at the highest pH (8.3) to achieve the most AMX recovery (see Figure 5). The results showed that increase in irradiation time (up to 60 min) improves AMX recovery performance of membrane without any sensible drop in membrane flux [14,37]. Furthermore it should be noted that longer irradiation time and hence excessive UV radiation on the membrane leads to surface degradation and rejection suppression.

FTIR analysis (Figure 7) confirms the presence of poly acrylic acid functional group (C=O and O-H) on the membrane surface. In spectra b, c and d, there are two bands: one at 3300 cm−1 assigned for (OH) and another large band at 1730 cm−1 due to (C=O) stretching of the carboxylic group. These bands which are absent or very week in the spectra of the original membranes (a), indicate successful photopolymerization. Moreover it is clear from the FTIR spectrum that for grafted membranes (b, c and d), an increase in irradiation time, is correlated to a decrease in absorbance peak intensity, due to increased AA deposition on the surface.

In cross section SEM images (Figure 8), Membrane structure conversion from finger like to sponge structure which is sign of delayed demixing. This variation is stronger for low molecular PEG membrane so that sponge structure involves more than half of membrane thickness in 60°C. It is worthy of respect that always there is a breakthrough point which dominant mechanism changes from thermodynamic factor to kinetic factor. This concentration in polysulfone membranes determined at 12% wt. for PEG additive [38] and 5% wt for PVP additive [39]. Here, in low concentration of additive in dope solution, the thermodynamic factor is predominant on kinetic factor in phase inversion process which cause spontaneous demixing and enlarge macrovoids (compare A2 and B2).

As studied by Bowen et al. (1999) the surface roughness parameter increases with an increase in pore size [40]. SEM surface images (Figure 9) showed the decrease in surface roughness with increasing in irradiation time which confirms the decreasing in membrane pore size.

## Conclusions

In this study, application of UV-grafted modified nanofiteration membrane for the separation of amoxicillin as a model pharmaceutical was investigated. The effect of various parameters such as molecular weight of PEG additive in polymeric solution, coagulation bath temperature, time of modification and pH of feed solution on membrane performance was investigated. The results can be summarized as follows:

Increase in PEG molecular weight resulted in decrease in rejection and increase permeability by pore enlargement, but in the other hand let monomers to enter pores easier and provide good condition for more effective modification.

Increase in coagulation bath temperature, due to enhancement of PEG solubility and coagulation time, led to smaller pores and improves separation performance.

With increase in modification time, pore size decreased and rejection increased. Also, membrane performance becomes more sensitive to pH. Application of very long irradiation time is not allowed due to damaging effect of UV radiation.

Increase in pH resulted in increasing membrane rejection due to electrostatic repulsion between solute and membrane. Also, solute molecules ability to get closer the membrane and deposit on surface decreased which resulted in increased flux.

Applying higher MW of PEG and lower CBTs deduced to fabricating membrane with larger pore size which is appropriate for UV irradiation and prepared membrane are of high permeability and AMX recovery.

Cross sectional SEM images confirmed pore size reduction and formation of sponge structure due delayed demixing due to coagulation bath temperature.

Surface SEM images showed a decrease in membrane surface roughness by increasing in irradiation time which according to literatures is corresponding to decrease in membarane pore size.

## Competing interests

The authors declare that they have no competing interests.

## Authors’ contributions

RD participated in the acquisition, analysis, and interpretation of data and helped to draft the manuscript; MH participated in the conception of the study, interpretation of data and in the given final approval of the version to be published; AA and MRM supervised the study in all steps (acquisition, analysis, and interpretation of data). All authors read and approved the final manuscript.
